# Correction: Synergistic activation of pro-inflammatory type-2 CD8 ^+^ T lymphocytes by lipid mediators in severe eosinophilic asthma

**DOI:** 10.1038/s41385-018-0121-5

**Published:** 2018-12-05

**Authors:** Bart Hilvering, Timothy S. C. Hinks, Linda Stöger, Emanuele Marchi, Maryam Salimi, Rahul Shrimanker, Wei Liu, Wentao Chen, Jian Luo, Simei Go, Timothy Powell, Jennifer Cane, Samantha Thulborn, Ayako Kurioka, Tianqi Leng, Jamie Matthews, Clare Connolly, Catherine Borg, Mona Bafadhel, Christian B. Willberg, Adaikalavan Ramasamy, Ratko Djukanović, Graham Ogg, Ian D. Pavord, Paul Klenerman, Luzheng Xue

**Affiliations:** 1 Respiratory Medicine Unit and Oxford Biomedical Research Centre, Nuffield Department of Medicine, John Radcliffe Hospital, University of Oxford, Oxford, UK; 2 Clinical and Experimental Sciences, University of Southampton Faculty of Medicine, Southampton University Hospital, Southampton, UK; 3 Translational Gastroenterology Unit and Peter Medawar Building, Nuffield Department of Medicine, University of Oxford, South Parks Rd, Oxford, UK; 4 MRC Human Immunology Unit, Weatherall Institute of Molecular Medicine, John Radcliffe Hospital, University of Oxford, Oxford, UK; 5 Transcriptomic Core Facility, The Jenner Institute, University of Oxford, Oxford, UK; 6 NIHR Southampton Respiratory Biomedical Research Unit, Southampton University Hospital, Southampton, UK

Correction to: *Mucosal Immunology* (2018) **11**: 1408–1419. 10.1038/s41385-018-0049-9

This article was originally published under standard licence, but has now been made available under a [CC BY 4.0] license. The PDF and HTML versions of the paper have been modified accordingly.

